# Supplementary material for: Association between altered cognition and Loa loa microfilaremia: First evidence from a cross-sectional study in a rural area of the Republic of Congo
Source: PLoS Negl Trop Dis. 2023 Jun 20;17(6):e0011430. doi: 10.1371/journal.pntd.0011430 (PMC10313009; doi:10.1371/journal.pntd.0011430)
Supplement: S1 Table — (DOCX) [file pntd.0011430.s001.docx]

|  | **Memory (/5)** | | **Language (/5)** | | **Visuospatial ability (/4)** | | **Orientation (/6)** | | **Attention (/6)** | | **Executive function (/2)** | | **Abstract reasoning (/2)** | |
| --- | --- | --- | --- | --- | --- | --- | --- | --- | --- | --- | --- | --- | --- | --- |
| **Variables** | aIRR [95% CI] | *P* | aIRR [95% CI] | *P* | aIRR [95% CI] | *P* | aIRR [95% CI] | *P* | aIRR [95% CI] | *P* | aOR [95% CI] | *P* | aOR [95% CI] | *P* |
| **Age (years old)** | | | | | | | | | | | | | | |
| 18-30 | Ref. |  | Ref. |  | Ref. |  | Ref. |  | Ref. |  | Ref. |  | Ref. |  |
| 41-50 | 0.41 [0.18, 0.93] | .032 | 0.98 [0.76, 1.25] | .873 | 0.86 [0.58, 1.27] | .453 | 0.96 [0.78, 1.18] | .700 | 0.90 [0.68, 1.21] | .498 | 1.89 [0.50, 7.21] | .348 | 1.88 [0.57, 6.18] | .299 |
| 51-60 | 0.40 [0.15, 1.08] | .070 | 0.99 [0.74, 1.31] | .938 | 1.02 [0.64, 1.62] | .935 | 0.88 [0.68, 1.13] | .305 | 0.75 [0.51, 1.08] | .124 | 1.09 [0.22, 5.49] | .912 | 1.44 [0.39, 5.31] | .581 |
| > 60 | 0.41 [0.15, 1.15] | .092 | 1.01 [0.75, 1.36] | .924 | 1.13 [0.70, 1.81] | .612 | 0.85 [0.65, 1.10] | .221 | 0.71 [0.48, 1.06] | .097 | 1.03 [0.17, 6.28] | .973 | 1.26 [0.33, 4.72] | .734 |
| **Sex** | | | | | | | | | | | | | | |
| Female | Ref. |  | Ref. |  | Ref. |  | Ref. |  | Ref. |  | Ref. |  | Ref. |  |
| Male | 0.59 [0.27, 1.33] | .207 | 1.26 [0.98, 1.62] | .070 | 2.28 [1.34, 3.88] | .002 | 0.96 [0.77, 1.19] | .723 | 1.43 [1.01, 2.03] | .042 | 0.81 [0.18, 3.57] | .778 | 0.87 [0.29, 2.59] | .805 |
| ***Loa* MFD (mf/mL)** | | | | | | | | | | | | | | |
| 0 | Ref. |  | Ref. |  | Ref. |  | Ref. |  | Ref. |  | Ref. |  | Ref. |  |
| 1-1999 | 0.58 [0.24, 1.45] | .247 | 0.97 [0.73, 1.28] | .816 | 1.33 [0.86, 2.05] | .199 | 0.92 [0.73, 1.18] | .528 | 0.84 [0.60, 1.18] | .315 | 0.52 [0.11, 2.43] | .406 | 0.60 [0.18, 1.97] | .403 |
| 2000-6,999 | 1.14 [0.50, 2.62] | .753 | 0.99 [0.76, 1.29] | .958 | 1.23 [0.78, 1.94] | .372 | 0.90 [0.71, 1.15] | .411 | 1.05 [0.76, 1.44] | .776 | 1.00 [0.24, 4.02] | .999 | 0.62 [0.21, 1.86] | .396 |
| 7000-14,999 | 1.08 [0.46, 2.57] | .853 | 1.12 [0.86, 1.45] | .406 | 1.39 [0.90, 2.12] | .133 | 1.01 [0.80, 1.27] | .946 | 0.79 [0.56, 1.12] | .187 | 0.12 [0.02, 0.65] | .014 | 1.71 [0.44, 6.66] | .439 |
| ≥ 15,000 | 0.28 [0.10, 0.77] | .014 | 0.99 [0.76, 1.29] | .953 | 1.00 [0.62, 1.61] | .993 | 0.95 [0.75, 1.20] | .687 | 0.55 [0.37, 0.82] | .003 | 0.16 [0.03, 0.88] | .036 | 3.14 [0.80, 12.18] | .098 |
| **History of cerebral malaria** | | | | | | | | | | | | | | |
| No | Ref. |  | Ref. |  | Ref. |  | Ref. |  | Ref. |  | Ref. |  | Ref. |  |
| Yes | 1.32 [0.47, 3.74] | .598 | 1.00 [0.73, 1.37] | .998 | 1.12 [0.64, 1.96] | .680 | 1.08 [0.82, 1.41] | .584 | 1.10 [0.73, 1.68] | .620 | 4.09 [0.62, 27.01] | .143 | 0.35 [0.08, 1.47] | .153 |
| **Presence of large artery atheroma** | | | | | | | | | | | | | | |
| No | Ref. |  | Ref. |  | Ref. |  | Ref. |  | Ref. |  | Ref. |  | Ref. |  |
| Yes | 0.76 [0.31, 1.81] | .531 | 0.78 [0.61, 1.01] | .060 | 1.01 [0.66, 1.53] | .977 | 1.00 [0.80, 1.24] | .976 | 0.97 [0.69, 1.34] | .840 | 0.15 [0.02, 1.05] | .056 | 1.15 [0.41, 3.24] | .787 |
| **Possible cerebral microangiopathy** | | | | | | | | | | | | | | |
| No | Ref. |  | Ref. |  | Ref. |  | Ref. |  | Ref. |  | Ref. |  | Ref. |  |
| Yes | 1.07 [0.22, 5.13] | .927 | 1.06 [0.72, 1.57] | .759 | 1.30 [0.64, 2.63] | .474 | 0.76 [0.51, 1.13] | .173 | 1.28 [0.75, 2.19] | .357 | 4.99 [0.46, 53.74] | .185 | 0.42 [0.08, 2.25] | .313 |
| MD | 1.17 [0.47, 2.89] | .740 | 1.13 [0.85, 1.51] | .391 | 1.00 [0.57, 1.75] | .997 | 1.03 [0.79, 1.33] | .820 | 0.92 [0.59, 1.44] | .726 | 1.03 [0.16, 6.49] | .975 | 0.95 [0.26, 3.52] | .946 |
| **Years of schooling** | | | | | | | | | | | | | | |
| In continuous | 1.14 [1.05, 1.25] | .003 | 1.02 [0.99, 1.04] | .185 | 1.19 [1.14, 1.25] | <.001 | 1.03 [1.01, 1.06] | .006 | 1.13 [1.09, 1.16] | <.001 | 1.64 [1.36, 1.98] | <.001 | 1.09 [0.96, 1.24] | .195 |
| **High blood pressure** | | | | | | | | | | | | | | |
| No | Ref. |  | Ref. |  | Ref. |  | Ref. |  | Ref. |  | Ref. |  | Ref. |  |
| Yes | 0.71 [0.34, 1.48] | .360 | 1.03 [0.83, 1.28] | .777 | 1.10 [0.74, 1.65] | .622 | 0.99 [0.81, 1.20] | .911 | 1.13 [0.83, 1.55] | .431 | 1.26 [0.32, 5.00] | .741 | 0.74 [0.28, 1.88] | .521 |
| **Use of smoking tobacco** | | | | | | | | | | | | | | |
| No | Ref. |  | Ref. |  | Ref. |  | Ref. |  | Ref. |  | Ref. |  | Ref. |  |
| Yes | 1.63 [0.78, 3.41] | .192 | 0.95 [0.76, 1.19] | .668 | 0.92 [0.63, 1.33] | .647 | 1.03 [0.85, 1.26] | .765 | 1.18 [0.90, 1.55] | .238 | 2.39 [0.63, 9.08] | .202 | 0.82 [0.29, 2.42] | .720 |
| ***Loa* Antibody rapid test** | | | | | | | | | | | | | | |
| Negative | Ref. |  | Ref. |  | Ref. |  | Ref. |  | Ref. |  | Ref. |  | Ref. |  |
| Positive | 0.98 [0.37, 2.58] | .961 | 1.01 [0.77, 1.32] | .936 | 1.19 [0.72, 1.95] | .498 | 0.93 [0.74, 1.17] | .536 | 1.19 [0.82, 1.74] | .373 | 2.18 [0.36, 13.13] | .394 | 1.51 [0.48, 4.69] | .474 |
| MD | 0.30 [0.05, 1.78] | .186 | 0.95 [0.58, 1.53] | .824 | 1.13 [0.50, 2.56] | .759 | 0.94 [0.62, 1.42] | .770 | 1.25 [0.68, 2.28] | .469 | 1.00 [NC] | .994 | 0.98 [NC] | .989 |
| Regression used | Negative binomial | | Poisson | | Poisson | | Poisson | | Poisson | | Ordinal | | Ordinal | |

Abbreviations: aIRR, adjusted Incidence Rate Ratio; CI, confidence intervals; aOR, adjusted Odds Ratio; MFD, microfilarial densities; NC, not calculable

**Table S1. Sub-items models (Binomial negative, Poisson or ordinal) with *L. loa* microfilarial densities in categories**
