# Supplementary material for: Association between altered cognition and Loa loa microfilaremia: First evidence from a cross-sectional study in a rural area of the Republic of Congo
Source: PLoS Negl Trop Dis. 2023 Jun 20;17(6):e0011430. doi: 10.1371/journal.pntd.0011430 (PMC10313009; doi:10.1371/journal.pntd.0011430)
Supplement: S2 Table — (DOCX) [file pntd.0011430.s002.docx]

| *L. loa* MFD (mf/mL) | **MoCA cut-offs** | | | | | |
| --- | --- | --- | --- | --- | --- | --- |
|  | **< 20/30** | | **< 21/30** | | **< 22/30** | |
|  | aOR^*^ [95% CI] | *P* | aOR^*^ [95% CI] | *P* | aOR^*^ [95% CI] | *P* |
| 0 | Ref. |  | Ref. |  | Ref. |  |
| 1-1999 | 2.06 [0.41, 10.42] | .383 | 1.34 [0.19, 9.34] | .770 | 1.12 [0.15, 8.14] | .913 |
| 2000-6,999 | 0.81 [0.17, 3.76] | .790 | 0.62 [0.10, 3.80] | .603 | 0.52 [0.07, 3.62] | .511 |
| 7000-14,999 | 0.91 [0.20, 4.19] | .901 | 1.42 [0.24, 8.42] | .696 | 1.62 [0.22, 11.61] | .633 |
| ≥ 15,000 | 6.66 [0.94, 46.97] | .057 | 17.54 [1.31, 234.09] | .030 | 11.00 [0.80, 152.02] | .073 |

^*^ adjusted on the same variables as in Table 3 (age, sex, history of cerebral malaria, high blood pressure, use of smoking tobacco, years of schooling, loiasis rapid antibody test, presence of large artery atheroma, possible cerebral microangiopathy)

**Table S2. Sensitivity analysis: adjusted logistic regression explain using different MoCA score cut-offs for the definition of altered cognition**
