# Supplementary material for: Association between altered cognition and Loa loa microfilaremia: First evidence from a cross-sectional study in a rural area of the Republic of Congo
Source: PLoS Negl Trop Dis. 2023 Jun 20;17(6):e0011430. doi: 10.1371/journal.pntd.0011430 (PMC10313009; doi:10.1371/journal.pntd.0011430)
Supplement: S3 Table — (DOCX) [file pntd.0011430.s003.docx]

| **General description of MoCA results** | | | | | | | |
| --- | --- | --- | --- | --- | --- | --- | --- |
| Variables | Mean | SD | Min | Max | 25^th^ Perc. | Median | 75^th^ Perc. |
| Total score (/30) | 15.64 | 5.81 | 3 | 28 | 11 | 16 | 20 |
| Memory (/5) | 1.27 | 1.77 | 0 | 5 | 0 | 0 | 3 |
| Language (/5) | 3.82 | 1.2 | 0 | 5 | 3 | 4 | 5 |
| Visuospatial ability (/4) | 1.38 | 1.44 | 0 | 4 | 0 | 1 | 3 |
| Orientation (/6) | 4.91 | 1.33 | 0 | 6 | 4 | 5 | 6 |
| Attention (/6) | 2.34 | 1.96 | 0 | 6 | 0 | 2 | 4 |
| Fluency (/12) | 4.34 | 3.63 | 0 | 12 | 0 | 4 | 8 |
| Abstract reasoning (/2) | 1.63 | 0.64 | 0 | 2 | 1 | 2 | 2 |
| Executive function (/2) | 0.3 | 0.57 | 0 | 2 | 0 | 0 | 0 |

| **Mean MoCA results by age categories (in years)** | | | | | | |
| --- | --- | --- | --- | --- | --- | --- |
| Variables | 18-40 (n=34) | 41-50 (n=40) | 51-60 (n=27) | > 60 (n=42) | Total (143) | *P^**^* |
| Total score (/30) | 18.7 (17.2 - 20.1) | 16.9 (15.5 - 18.4) | 14.1 (11.8 - 16.4) | 13.0 (11.0 - 14.9) | 15.6 (14.7 - 16.6) | .078 |
| Memory (/5) | 2.0 (1.3 - 2.7) | 1.2 (0.7 - 1.7) | 1.1 (0.5 - 1.7) | 0.8 (0.3 - 1.3) | 1.3 (1.0 - 1.6) | .025 |
| Language (/5) | 4.0 (3.7 - 4.3) | 4.0 (3.6 - 4.3) | 3.6 (3.1 - 4.1) | 3.7 (3.3 - 4.1) | 3.8 (3.6 - 4.0) | .368 |
| Visuospatial ability (/4) | 1.8 (1.4 - 2.3) | 1.4 (1.0 - 1.7) | 1.3 (0.7 - 1.9) | 1.1 (0.6 - 1.6) | 1.4 (1.1 - 1.6) | .190 |
| Orientation (/6) | 5.5 (5.2 - 5.8) | 5.4 (5.1 - 5.7) | 4.5 (4.0 - 5.1) | 4.2 (3.7 - 4.6) | 4.9 (4.7 - 5.1) | < .001 |
| Attention (/6) | 3.3 (2.7 - 3.9) | 2.8 (2.2 - 3.3) | 1.8 (1.1 - 2.6) | 1.5 (0.9 - 2.1) | 2.3 (2.0 - 2.7) | < .001 |
| Fluency (/12) | 5.4 (4.4 - 6.5) | 5.5 (4.3 - 6.7) | 3.4 (2.2 - 4.7) | 3.0 (1.9 - 4.0) | 4.3 (3.7 - 4.9) | < .001 |
| Abstract reasoning (/2) | 1.6 (1.4 - 1.8) | 1.8 (1.6 - 1.9) | 1.6 (1.4 - 1.9) | 1.5 (1.3 - 1.7) | 1.6 (1.5 - 1.7) | .336 |
| Executive function (/2) | 0.4 (0.2 - 0.6) | 0.5 (0.2 - 0.7) | 0.2 (0.1 - 0.4) | 0.1 (0.0 - 0.3) | 0.3 (0.2 - 0.4) | .064 |

| **Mean MoCA results by sex** | | | |
| --- | --- | --- | --- |
| Variables | Female (n=47) | Male (n=96) | *P^*^* |
| Total score (/30) | 12.3 (10.9 - 13.8) | 17.3 (16.1 - 18.4) | <.001 |
| Memory (/5) | 1.1 (0.6 - 1.6) | 1.3 (1.0 - 1.7) | .454 |
| Language (/5) | 3.3 (2.9 - 3.7) | 4.1 (3.9 - 4.3) | < .001 |
| Visuospatial ability (/4) | 0.4 (0.2 - 0.7) | 1.8 (1.6 - 2.1) | < .001 |
| Orientation (/6) | 4.5 (4.1 - 4.9) | 5.1 (4.8 - 5.4) | .017 |
| Attention (/6) | 1.2 (0.7 - 1.7) | 2.9 (2.5 - 3.3) | < .001 |
| Fluency (/12) | 2.4 (1.5 - 3.3) | 5.3 (4.6 - 6.0) | < .001 |
| Abstract reasoning (/2) | 1.6 (1.4 - 1.8) | 1.7 (1.5 - 1.8) | .472 |
| Executive function (/2) | 0.2 (0.0 - 0.3) | 0.4 (0.2 - 0.5) | .055 |

| **Mean MoCA results by *Loa* microfilaremic status (negative/positive) and MFD categories (mf/mL)** | | | | | | | | | |
| --- | --- | --- | --- | --- | --- | --- | --- | --- | --- |
| Variables | Negative (n=47) | Positive (n=96) | *P^*^* | 1-1999 (n=22) | 2000-6999 (n=25) | 7000-14,999 (n=24) | ≥ 15,000 (n=25) | *P^**^* | *P^***^* |
| Total score (/30) | 16.3 (14.6 - 17.9) | 15.3 (14.2 - 16.5) | .380 | 16.0 (13.6 - 18.5) | 14.6 (11.9 - 17.3) | 17.0 (14.6 - 19.3) | 13.6 (11.4 - 15.7) | .249 | .214 |
| Memory (/5) | 1.5 (0.9 - 2.0) | 1.2 (0.8 - 1.5) | .341 | 1.2 (0.4 - 2.0) | 1.4 (0.7 - 2.1) | 1.6 (0.7 - 2.4) | 0.5 (0.0 - 1.0) | .208 | .098 |
| Language (/5) | 3.8 (3.4 - 4.1) | 3.8 (3.6 - 4.1) | .716 | 3.7 (3.1 - 4.4) | 3.5 (2.9 - 4.1) | 4.3 (3.9 - 4.6) | 3.8 (3.4 - 4.2) | .344 | .542 |
| Visuospatial ability (/4) | 1.2 (0.8 - 1.6) | 1.5 (1.2 - 1.8) | .264 | 1.8 (1.2 - 2.4) | 1.2 (0.6 - 1.9) | 1.7 (1.0 - 2.3) | 1.1 (0.6 - 1.7) | .335 | .979 |
| Orientation (/6) | 5.1 (4.7 - 5.4) | 4.8 (4.6 - 5.1) | .331 | 4.9 (4.4 - 5.4) | 4.4 (3.7 - 5.2) | 5.3 (4.9 - 5.7) | 4.7 (4.2 - 5.3) | .179 | .597 |
| Attention (/6) | 2.7 (2.1 - 3.2) | 2.2 (1.8 - 2.6) | .168 | 2.5 (1.7 - 3.3) | 2.3 (1.4 - 3.1) | 2.3 (1.4 - 3.1) | 1.4 (0.7 - 2.2) | .141 | .015 |
| Fluency (/12) | 4.4 (3.3 - 5.4) | 4.3 (3.6 - 5.1) | .965 | 5.0 (3.4 - 6.6) | 3.7 (2.3 - 5.0) | 5.1 (3.8 - 6.4) | 3.4 (1.8 - 5.0) | .456 | .490 |
| Abstract reasoning (/2) | 1.7 (1.5 - 1.8) | 1.6 (1.5 - 1.7) | .500 | 1.5 (1.2 - 1.8) | 1.4 (1.1 - 1.7) | 1.7 (1.4 - 2.0) | 1.8 (1.5 - 2.0) | .303 | .289 |
| Executive function (/2) | 0.4 (0.2 - 0.6) | 0.2 (0.1 - 0.3) | .067 | 0.3 (0.0 - 0.6) | 0.3 (0.1 - 0.5) | 0.2 (0.0 - 0.3) | 0.2 (-0.0 - 0.3) | .273 | .028 |

^*^ P-values by t-test; ^**^ P-values by ANOVA ; ^***^ P-values by Trend test of Cuzick

Abbreviations: SD, Standard deviation; Min, Minimum; Max, Maximum; Perc. Percentile

**Tables S3**. MoCA scores by age, sex, *Loa* microfilaremic status and *L. loa* MFD
