# Supplementary material for: Association between altered cognition and Loa loa microfilaremia: First evidence from a cross-sectional study in a rural area of the Republic of Congo
Source: PLoS Negl Trop Dis. 2023 Jun 20;17(6):e0011430. doi: 10.1371/journal.pntd.0011430 (PMC10313009; doi:10.1371/journal.pntd.0011430)
Supplement: Attachment — Submitted filename: Response_PLOSNTD_Final.docx [file pntd.0011430.s006.docx]

**Dr. J.R. Zunt, Academic Editor
 Dr F. Tamarozzi, Section Editor PLOS Neglected Tropical Diseases**

Paris, May 31^th^ 2023.

**RE: Manuscript PNTD-D-23-00393**

**“Association between altered cognition and *Loa loa* microfilaremia: First evidence from a cross-sectional study in a rural area of the Republic of Congo”**

Dear Drs. Zunt and Tamarozzi,

Thank you for inviting us to revise and resubmit the above-mentioned manuscript. We have made the revisions according to the reviewers’ comments and are grateful for these thoughtful remarks. You will find below detailed comments about the way we handled each item in the revised version.

We mainly added the limitations raised by the reviewers in a short section in the discussion, and stressed the needed for further confirmative studies on our findings, which planning is underway by our team.

We are looking forward to your further thoughts on this manuscript along with those of the reviewers. All the authors have been involved with the revisions and agree with them.

Sincerely,

**Dr Jérémy T. Campillo**

# REVIEWER #1

## The objectives are clearly articulated with clear testable hypothesis. The study design is appropriate to address the stated objectives, but not clearly specified as historical cohort study. The population is clearly described and appropriate for the hypothesis being tested. The sample size was not clearly calculated, but this is the first time such study is conducted. It would have been difficult to calculate the sample size without at least some preliminary results. The statistical analysis is appropriate. There are no concerns about ethical or regulatory requirement. They are all met.

We thank the reviewer #1 for his comments. We clarified in the manuscript that our study is nested in the prospective “MorLo” cohort in Congo. We agree that the sample size was not easy to calculate as preliminary data were missing on this topic. We chose to recruit the maximum number of subjects with high MFD, and match them with one individual with low MFD, and one amicrofilaremic individual.

To clarify this, we have added the following sentences in the methods section on page 6: *“To investigate neurological morbidity, we conducted this pilot cross-sectional study nested in the cohort. Three groups were constituted within the whole cohort, to ensure adequate comparability. Each individual with high L. loa microfilarial density (MFD) (> 8000 mf/mL) was matched on sex, age (± 5 years) and village of residence with two others: one subject with low L. loa MFD (500 to 7999 mf/mL) and one amicrofilaremic individual. “*

## The results are clearly and completely presented. The tables are appropriate.

We thank the reviewer #1 for this thoughtful comment.

## The conclusions are supported by the data presented. The limitation of the analysis is described but not complete. No recommendation was made concerning further exploration in the domain despite the doubts concerning the results of the present study.

We agree that further exploration is needed to better understand the relationship between cognitive impairment and loiasis, as well as a potential mechanism. We aim to further investigate this relationship using large sample size, validated comprehensive cognitive evaluation for MCI and dementia, and long-term follow-up. As this comment is particularly relevant, we have highlighted the need for further exploration in both the abstract and the discussion.

We have modified the abstract (page 2) and the discussion (page 19)

*“Further studies investigating neurological morbidity of loiasis are needed. “*

*“To address these limitations, larger studies with comprehensive and validated cognitive testing, long term follow–up,* *brain imaging and adjustment for coinfections are currently under planning. […] Our results provide the rationale for further studies investigating consideration of the impact of L. loa* *microfilaremia on cognitive function.”*

## Page 6: “Study data were provided to community health workers in the involved villages to inform the local community of the study results and patients of their test results.” I will suggest the results of the study were shared with the local health personnel to explain to the community members, and the individual results of the patients were put at their disposal through the local health personnel.

We agree with reviewer #1 on the role of health community workers from each village to share biological and medical results. Test results were available for patients after the completion of the study to ensure both patient and health workers were blinded to the parasitological results during the completion of the study.

We have rephrased the methods section to clarify this on page 6-7 *“The main results of the study were provided to community health workers in the involved villages. For each patient, the results of their tests were safely and confidentially kept at the health facility of their village and were accessible upon request.*

## Page 8: “The results of MoCA by a neurologist blinded to the parasitological results.” It is not mentioned if the trained nurses examined the patients blinded of parasitological results.

The trained nurses in charge of the MoCA testing were also blinded to the parasitological results, as well as the patients at the time of the study.

We have modified the methods section to clarify this on page 7-8 *“Nurses were blinded to the parasitological results”.*

## The paper by Checkouri et al on the “association between altered cognition and Loa loa microfilaremia: first evidence from a cross-sectional study in a rural area of the Republic of Congo” is an important contribution of the impact of loiasis, the neglected among the neglected by the scientific community. It is the first time the neuropsychiatric complications are described particularly in subject harboring very high MFD. In the logistic regression models and the Poisson regression models, there is no dose effect for the relation between the high MFD and the altered cognition statute. This put some doubt on this relation and as this has never been demonstrated elsewhere adding to possible confounding factors, the authors should clearly recommend the conduct of similar studies in different settings to confirm or to refute this relationship between the high loa MFD and the altered cognition.

Indeed, we did not find a dose effect in our statistical analyses, we found a relationship between the highest microfilaremic loads (> 15,000 mf/mL) and impaired cognition. It is possible that the effect found only exists from a certain "dose" (intensity) of infection or that the power of the study did not enable to demonstrate this dose effect. Additional studies (currently underway) will likely elucidate this issue.

We have modified the discussion section to clarify this on page 17 *“Although the significant association between alteration of cognition and loiasis was observed in patients with high MFD, we observed no clear dose–effect of L. loa MFD on MoCA score. This may reflect a threshold effect at which the neurocognitive effect would appear, or a lack of power due to sample size. Further studies will help to better understand this phenomenon and to evaluate the validity of this MoCA in rural areas of Central Africa”.*

Furthermore, we agree that there should be other studies to investigate the relationship between loiasis and cognitive impairment, allowing us to have a larger sample size, confirmation of cognition alteration and possibly explanation of the causative mechanism. This has been highlighted in both the abstract and in the discussion.

We have modified the abstract (page 2) and the discussion (page 19)

*“Further studies investigating neurological morbidity of loiasis are needed. “*

*“To address these limitations, larger studies with comprehensive and validated cognitive testing, long term follow–up, brain imaging and adjustment for coinfections are currently under planning […] Our results provide the rationale for further studies investigating consideration of the impact of L. loa microfilaremia on cognitive function.”*

# REVIEWER #2

## The objectives of the study are clearly articulated, the design is appropriate, given the study context, the population is well described, the sample size is sufficient, even though not justified. The statistical methods used are correct to support the conclusions. The study received ethical clearances from regulatory instances.

We thank the reviewer #2 for her/his comment. We were not able to calculate the sample size before the study given the lack of preliminary data on this topic. We chose to recruit the highest number of patients with high MFD, and match them 1:1:1 with patients with low MFD, and amicrofilaremic patients.

## The analyses presented match the analysis plan and the results are clearly and completely presented. Tables and figures are of good quality and clarity.

We thank the reviewer #2 for this thoughtful comment.

## The conclusions are supported by the data presented. the limitations of the analysis are described, but more description need to be added to what is presented. The public health relevance is clearly addressed

Following the reviewer’s comment, we added in the discussion a paragraph mentioning the limitations of this study. Please note that the limitations are described further in our next response.

## The authors of this manuscript present for the first time an association between high density of Loa loa microfilariae in the peripheric blood and the alteration of cognition in a population living in endemic area of loiasis, independent of antifilarial treatment. The findings from this pilot study constitute a breakthrough for this very neglected tropical disease (Loiasis) and deserve to be shared with scientific community working in this field of research. The authors should highlight the fact that the study design they used “Cross-sectional” is the weakest design to establish causal relationship between exposure and disease in etiologic epidemiology. The good thing is that this study is nested in the cohort set up in a more ambitious project “Morbidity due to Loiasis” and one can expect that the findings from the long term follow up of participants would validate the preliminary observations.

We agree that such design does not allow to draw definite conclusion on cognitive alteration. These limitations have been added to the manuscript, and we plan to address the small sample size and the need for validation from complete cognitive testing with further studies. The cohort design will allow us to follow a possible cognitive decline over the years in each *Loa* infected individual using MoCA score (See for example Schrag A, Lancet Neurol 2017 in Parkinson’s disease).

We added this section to the discussion (page 19) *“Our study has several limitations. The cross–sectional design is not sufficient to establish a causal relationship between L. loa infection and neurocognitive disease. The lack of normative values for the screening test used in our population, as well as the relatively small sample size clearly limits the interpretability of our results. These limitations are inherent to the “pilot-study” design aimed to identify potential neurological morbidity in a disease which is largely considered as benign. To address these limitations, larger studies with comprehensive and validated cognitive testing, long term follow–up, brain imaging and adjustment for coinfections are currently under planning”*

# REVIEWER #3

## The MOCA is a screening test that may overestimate the frequency of cognitive impairment. This is more likely when the cut-off is not based on local norms of the study area. The study should have used the 50 individuals with MF to get their mean score and then generate z scores to identify those who scores fall below 2 standard deviations of the control group mean score.

We agree with the reviewer #3 that the use of MoCA as a screening tool may overestimate the frequency of cognitive impairment. As reported in the study by Thomann et al, the sample size needed to estimate a reliable Z-score in a new population for MoCA would be higher than 200 healthy individuals (See Thomann AE, J Alzheimers Dis. 2018; PMID: 29945351). This number was not available in our study (only 47 individuals with no microfilaremia), as our sample size was determined based on the number of patients with high MFD in the cohort at the time of the pilot study. Furthermore, the distribution of MoCA scores is not Gaussian and the proportion of patients with less than 4 years of school attendance leads to a floor effect. The use of Z-scores in such small population with heterogeneous schooling has several limitations which would have precluded us from the proper conclusions (See Toffalini et al., J Intellect Disabil Res. 2019 for the theoretical framework on this question). To address these limitations, we chose to use two approaches: A traditional approach using a lower cut-off value, as described in culturally heterogenous settings [Carson N et al. – Reference #16], and analyses using the MoCA score as a continuous variable, as suggested elsewhere [e.g. Yang C. et al. and Pugh EA et al. – References #17 and 18]. Both our analyses were coherent with a specific alteration of cognition in individuals with high MFD. Furthermore, we performed additional sensitivity analyses using different cut-offs for the definition of impaired cognition (20, 21 and 22/30), which all found statistically significant or borderline significant association with MoCA scores and high *Loa* MFD (See the statistical analysis on our next answer). Further studies are under planning and will be dedicated to the development of normative values of MoCA in this population with validation from comprehensive cognitive testing.

This is addressed in the new version of the Methods section (page 9) *“Using Poisson regression, MoCA score was analyzed as a continuous score to address considerable variation compared to the normative MoCA values developed in high income countries. Indeed, the lack of previously published normative values in similar settings (rural population of Central Africa), the relatively small sample size and the non-normal distribution of MoCA scores did not allow the use of Z–scores derived from the group with no microfilaremia.”*

## The three groups are not well described in the methods. More details are needed.

Following the reviewer’s comment, we further describe the 3 groups of this cross-sectional study with the following matching process: Each patient in the high MFD category from the cohort was matched 1:1:1 to one subject with low MFD, and one amicrofilaremic individual. The matching variables included age, sex and village, to ensure imbalance between these confounders.

This following alteration has been added to the Methods section (page 6) *“To investigate neurological morbidity, we conducted this pilot cross-sectional study nested in the cohort. Three groups were constituted within the whole cohort, to ensure adequate compatibility. Each individual with high L. loa microfilarial density (MFD) (> 8000 mf/mL) was matched on sex, age (± 5 years) and village of residence with two others: one subject with low L. loa MFD (500 to 7999 mf/mL) and one amicrofilaremic individual.”*

## I find the frequency of impairment (>80%) too high to be true....and this is probably due how they determined impairment. A better method has been suggested above.

We agree with the reviewer #3 that the frequency of impairment is probably related to the cut-off used and the screening nature of MoCA testing. The overestimation bias has been addressed in our previous answer. Moreover, we performed sensitivity analyses using different cut-offs (see below) for the definition of altered cognition (< 20, 21 and 22/30). These analyses using different MoCA cut-offs were consistent with our findings, with a significant (or borderline significant) association found with high levels of Loa MFD for each cut-off. However, these results are exploratory and subject to the above-mentioned bias, and therefore not mentioned in the manuscript. If the reviewer 3 thinks that this sensitivity analysis is useful, we could include the Table as Supplementary material.

| *L. loa* MFD (mf/mL) | **< 20/30** | | **< 21/30** | | **< 22/30** | |
| --- | --- | --- | --- | --- | --- | --- |
|  | aOR^*^ [95% CI] | *P* | aOR^*^ [95% CI] | *P* | aOR^*^ [95% CI] | *P* |
| 0 | Ref. |  | Ref. |  | Ref. |  |
| 1-1999 | 2.06 [0.41, 10.42] | .383 | 1.34 [0.19, 9.34] | .770 | 1.12 [0.15, 8.14] | .913 |
| 2000-6,999 | 0.81 [0.17, 3.76] | .790 | 0.62 [0.10, 3.80] | .603 | 0.52 [0.07, 3.62] | .511 |
| 7000-14,999 | 0.91 [0.20, 4.19] | .901 | 1.42 [0.24, 8.42] | .696 | 1.62 [0.22, 11.61] | .633 |
| ≥ 15,000 | 6.66 [0.94, 46.97] | .057 | 17.54 [1.31, 234.09] | .030 | 11.00 [0.80, 152.02] | .073 |

^*^ adjusted on the same variables as in Table 3 (age, sex, history of cerebral malaria, high blood pressure, use of smoking tobacco, years of schooling, loiasis rapid antibody test, presence of large artery atheroma, possible cerebral microangiopathy)

**Sensitivity analysis: adjusted logistic regression explain using different MoCA score cut-offs for the definition of altered cognition**

Along with other alterations, we added this sentence in the discussion (page 17) *“We used MoCA testing, a screening tool for MCI and dementia mainly used in high-income countries and not validated for the specific population investigated. The majority of patients had a positive screening for cognitive impairment (defined by a MoCA score < 23), probably related to the fact that normative values of MoCA are different in rural Congo.”*

## The results should be presented for the 3 different groups (high and low MF and the no MF group). How do the sociodemographic variables compared across the groups (Table 1) and MOCA scores (table 2).

We provide a new version of Table 2 in the manuscript, which describes sociodemographic, ultrasound and neurocognitive data from the 3 groups. As the matching process was designed to have comparable measures between these 3 groups, there were no difference in any of the baseline sociodemographic variables.

In order to improve the presentation of the results, table 1 and 2 have been interleaved. The new version of Table 1 is found on the manuscript (page 11).

## The discussion and conclusion should mention the limitations mentioned above (use of a screening test that may overestimate the impairment rates)

We agree with the reviewer #3 about the limitations mentioned. The new version of the manuscript provides a limitation section with these clearly addressed.

We added this section to the discussion (page 19) *“Our study has several limitations. The cross–sectional design is not sufficient to establish a causal relationship between L. loa infection and neurocognitive disease. The lack of normative values for the screening test used in our population, as well as the relatively small sample size clearly limits the interpretability of our results. These limitations are inherent to the “pilot-study” design aimed to identify potential neurological morbidity in a disease which is largely considered as benign.”*

## The authors in attempting to explain how MF lead to cognitive impairment could consider the BBB integrity being compromised by proinflammatory cytokines in response to infection. Future studies could consider investigating that.

We thank the reviewer #3 for her/his comment. Indeed, preliminary work from the 1950s (Van Bogaert et al., and Kivits et al.) as well as other works from Akué et al. have investigated this hypothesis. The first reference from Van Bogaert [41] has been added in the manuscript.

We added this hypothesis in our discussion (page 18) *“. Disruption of the blood–brain barrier by dead microfilariae or proinflammatory cytokines has also been suggested as a mechanism in historical cases of spontaneous encephalopathies [41].”*

*“Other possible mechanisms are related to the presence of the parasite in the cerebrospinal fluid or in the subdural space [37–40], or disruption of the blood–brain barrier [41]. […] Further investigation on the mechanism of cognitive alteration from loiasis* *would require brain MRI, which is not available in rural Congo, or lumbar puncture, an invasive procedure with no guaranteed benefit/risk ratio. “*

## The authors designed an exploratory study to examine the association between Loa loa infection and cognitive functioning. Challenges observed are the use of a screening test and not using local norms to determine impairment which led to an unusually high rate of impairment

We agree with the limitations raised by the reviewer #3. The revisions mentioned above have been made to the manuscript to further describe the limitations of our pilot study. Our study was aimed to “screen” for potential neurological alteration in a very neglected disease, affecting particularly people living in rural areas with no access to neurological examination. Please note that further studies on the topic are currently under planning, using appropriate sample size and validated testing, allowing us to strengthen our findings.

We have modified the abstract and the discussion to temper our findings (page 18)

*“Further studies investigating neurological morbidity of loiasis are needed.”*

*“These limitations are inherent to the “pilot-study” design aimed to identify potential neurological morbidity in a disease largely considered benign. To address these limitations, larger studies with comprehensive and validated cognitive testing, long term follow–up and brain imaging are currently under planning.*
